# Greater Acute Concussion Symptoms Are Associated With Longer Recovery Times in NCAA Division III Collegiate Athletes

**DOI:** 10.3389/fneur.2021.801607

**Published:** 2022-01-21

**Authors:** Grant L. Iverson, Douglas P. Terry, Bruce Maxwell, Ross Zafonte, Paul D. Berkner, Nathan E. Cook

**Affiliations:** ^1^Department of Physical Medicine and Rehabilitation, Harvard Medical School, Boston, MA, United States; ^2^MassGeneral Hospital for Children Sports Concussion Program, Boston, MA, United States; ^3^Department of Physical Medicine and Rehabilitation, Spaulding Rehabilitation Hospital, Charlestown, MA, United States; ^4^Spaulding Research Institute, Charlestown, MA, United States; ^5^Department of Computer Science, Colby College, Waterville, ME, United States; ^6^Department of Physical Medicine and Rehabilitation, Spaulding Rehabilitation Hospital, Massachusetts General Hospital, Brigham and Women's Hospital, Harvard Medical School, Boston, MA, United States; ^7^Home Base, A Red Sox Foundation and Massachusetts General Hospital Program, Boston, MA, United States; ^8^Department of Family Medicine, College of Osteopathic Medicine, University of New England, Biddeford, ME, United States

**Keywords:** concussion, athletes, sports, school, outcome, academics

## Abstract

We examined the association between the severity of acute concussion symptoms and time to return to school and to sports in National Collegiate Athletic Association (NCAA) Division III collegiate athletes. We hypothesized that students with the lowest burden of acute symptoms, measured in the first 72 h, would have the fastest return to school and sports and those with the highest burden of symptoms would have the slowest return to school and sports. This injury surveillance cohort included 808 athletes from 11 NCAA Division III colleges who sustained a concussion between 2014 and 2019. Athletic trainers documented time to return to school and to sports. Kruskal-Wallis tests with *post-hoc* planned comparison Mann-Whitney U tests were used to assess whether athletes took longer to return based on their acute symptom burden (Low, Medium, or High). Survival analysis (Kaplan Meier with log rank tests) was used to compare the recovery times based on acute symptom burden (censored at 28 days). Chi-square tests compared the proportion of those who had not yet returned to school or sports at various recovery benchmarks (i.e., 1 week, 10 days, 2 weeks, 3 weeks, 4 weeks) based on acute symptom burden. Women (median = 5 days) took slightly longer than men (median = 4 days) to return to school (*p* = 0.001; *r* = −0.11, small effect). Women and men did not differ on time to return to sports (*p* = 0.32, *r* = −0.04). A greater proportion with high acute symptoms remained out of school at 5 (odds ratio, OR = 4.53), 7 (OR = 4.98), and 10 (OR = 4.80) days compared to those with low acute symptoms. A greater proportion with high acute symptoms remained out of sports at 10 (OR = 4.11), 14 (OR = 3.46), and 21 (OR = 3.01) days compared to those with low acute symptoms. This study shows a strong association between having a high burden of acute post-concussion symptoms and having a slower return to school and sports in Division III collegiate athletes. Moreover, it also illustrates the converse: that those athletes with a low burden of acute symptoms have a faster return to school and sports.

## Introduction

The large majority of athletes who sustain a sport-related concussion will recover, from a clinical perspective, in <1 month (1–4). Physiological recovery, however, might take longer than clinical recovery (5). Athletes who experience greater symptoms in the first 72 h following a sport-related concussion are at risk for worse clinical outcome (6). This is intuitive because greater acute symptom reporting might reflect the *combined and amplifying effects* of the injury on neurobiology, adverse acute psychological reactions, and greater pre-injury propensity toward experiencing symptoms. The converse of this is logical, but it has not been well-studied: that those with a *low* burden of acute symptoms have *better* clinical outcome. This has not been explicitly studied to our knowledge, but can be inferred based on studies that show greater initial symptoms are associated with a protracted recovery (6), such as longer self-reported post-concussion symptom duration (7) and taking more than 10 days to be deemed recovered (8), as well as the finding that those with 5–7 and 8–13 acute symptoms following a concussion were more likely to take 10 or more days to return to play than those who only had 0–3 acute symptoms (9).

Using diverse samples and methodologies, researchers have reported an association between greater acute concussion symptoms and worse clinical outcome in high school students (8, 10, 11), college students (4, 12–14), professional athletes (15–17), and youth presenting to the emergency department (18, 19) or to specialty clinics (20–23). Researchers have defined and measured outcome in different ways, such as subjectively experienced symptoms (7, 8, 10–14, 18–20, 22–25), cognitive functioning (8, 25), balance (25), return to school/work (26, 27), and return to sports (4, 10, 15–17, 28–30). One of the most important, and essential, outcomes for injured student athletes is returning fully to school (31, 32). Studying this outcome, however, is very uncommon in the sport concussion literature (33).

The purpose of this study is to document the time it takes for concussed collegiate athletes to return to school, fully, without accommodations and to return to sports. A substantial number of student athletes participate in National Collegiate Athletic Association (NCAA) Division III championship sports. For example, during the 2018–2019 academic year, 193,814 college students, from 8,257 teams, participated in NCAA Division III championship sports (34). The data for this study were obtained from an injury surveillance program deployed to 11 NCAA Division III schools over the course of 5 consecutive academic years. We hypothesized that there would be an association between the severity of acute symptoms experienced in the first 72 h after concussion and functional outcome. Specifically, we hypothesized that those students with the lowest burden of acute symptoms would have the fastest return to school and sports and those with the highest burden of symptoms would have the slowest return to school and sports.

## Materials and Methods

### Design and Participants

This prospective naturalistic observational cohort study monitored the recovery of collegiate student athletes from 11 NCAA Division III colleges who sustained a sport-related concussion between 2014 and 2019 (5 academic years). Athletic trainers prospectively monitored concussion recovery using the Head Injury Tracker (HIT), a free injury surveillance application created by the Maine Concussion Management Initiative. In the first year the HIT was deployed to five colleges and by the final year it was used in 11 colleges. For the 2018–2019 academic year, the total undergraduate enrollment (all students, not only athletic participants) at each college ranged from 1,828 to 5,643, with a total undergraduate enrollment of 26,760.

The HIT injury surveillance database includes 1,005 college student athletes ages 17–27 who sustained a concussion between November 2014 and May 2019. Concussions were identified by the medical staff at the colleges, sometimes in collaboration with the students' other health care providers. From this original sample, 183 athletes were excluded (18.2% of total sample) [134 were excluded because they were not assessed within 3 days of injury, and 44 were excluded because dates were missing for both return to school and return to sports. Another five were excluded because of problematic dates that, due to data deidentification procedures, could not be confirmed/checked for accuracy and had a high probability of being data entry errors]. Lastly, there were 14 athletes (2% of the remaining sample) with outlier values on days to return to sports (>90 days; median 157 days) who were excluded. Sensitivity analyses including these 14 outliers revealed no significant change in the results from our analyses or effect size estimates.

The final sample included 808 student athletes (80.4% of the total cohort) between the ages of 17 and 27 (*M* = 20.4, *SD* = 1.4 years). The sample was almost equally split between women (*n* = 341, 42.2%) and men (*n* = 466, 57.7%), and one student athlete (0.1%) self-identified as other gender. For young women, the sports played at the time of injury were ice hockey (20.5%), rugby (17.6%), soccer (14.4%), lacrosse (9.1%), basketball (7.6%), volleyball (6.5%), and several other sports (24.3%). For young men, the sports played at the time of injury were football (37.3%), lacrosse (15.5%), ice hockey (12.4%), rugby (11.6%), soccer (8.8%), and several other sports (14.4%).

### Measures

#### Demographic, Health History, and Injury Information

Demographic and self-reported health history information was collected from the student athletes. Injury information collected by an athletic trainer included the concussion date and the scenario in which the concussion occurred (e.g., team vs. not team activities; practice vs. game).

#### Concussion Symptoms

Athletes completed the Post-Concussion Symptom Scale (PCSS), a self-report questionnaire that includes 22 symptoms rated from zero to six in terms of severity, with 1 or 2 reflecting “mild,” 3 or 4 reflecting “moderate,” and 5 or 6 reflecting “severe” problems with a given symptom. The total symptom severity score (range 0–132) was analyzed in this study. Acute PCSS scores (completed within 3 days of injury) were used to divide the athletes into the following three groups, representing approximately one-third of the total sample in each group (cut-offs at the 33rd and 66th percentiles): Low Acute Symptom Severity (PCSS score 0 through 9), Medium Acute Symptom Severity (10 through 22 for women and 10 through 23 for men), and High Acute Symptom Severity (23 or greater for women and 24 or greater for men).

#### Recovery Time

Athletic trainers entered the date students returned to school (full time without accommodations) and the date they returned to sports (completed return to play protocol) following their concussion. Recovery time was calculated as the number of days between the injury date and the date of return to school and the date of return to sports.

### Procedures

The HIT application is an online injury surveillance platform. When a student athlete sustained a concussion, an athletic trainer entered information about the injury and the athlete's recovery via smartphone or webpage and collected the athlete's symptom questionnaire responses (PCSS). All athletic trainers/school officials involved in data collection completed an online training about the HIT application format and the process for entering information. Student athletes and schools were not compensated for their participation. Technical assistance and data collection oversight was provided by a dedicated HIT project coordinator. Institutional review board approval for the creation of the deidentified database and use of it for research was obtained.

### Statistical Analyses

Demographic differences between groups and potential associations between demographic variables and the outcome variables were assessed via Mann-Whitney U (M–W U) tests, ANOVA, Spearman rho correlation coefficients, and chi-square tests. The two outcome variables (days to return to school and days to return to sports) were non-normally distributed (Shapiro-Wilk *p*-values < 0.05), so Kruskal-Wallis tests were employed to assess whether athletes took longer to return based on their acute symptom burden (Low, Medium, or High). For statically significant Kruskal-Wallis (K-W) tests, *post-hoc* planned comparison M-W U tests were conducted. Survival analysis (Kaplan Meier with log rank tests) was used to compare the recovery times based on acute symptom burden (censored at 28 days). To maximize the clinical relevance, each athlete's return to school/sports status was also dichotomized (i.e., returned or not) at various recovery benchmarks (i.e., 3 and 5 days for school return, and 7-, 10-, 14-, 21-, and 28-days for return to school and return to sports) and chi-square tests, with corresponding odds ratios (OR) as measures of effect size, were conducted to compare the proportion of those who had not yet returned to school or sports at these various time points based on acute symptom burden. There were 16 primary statistical tests (2 K-W tests, 2 log rank tests, 12 chi-square tests); we used a Bonferroni correction to maintain a family wise error rate of 5% and thus, we set alpha at 0.003 (0.05/16). Cumulative recovery curves were constructed to visually display the proportion of student athletes who returned to school or sports over time. The M-W U test Z-values were used to calculate a non-parametric effect size *r*, where *r* = $\frac{Z}{\sqrt{}N}$ (35), which was interpreted according to conventional guidelines, i.e., *r* = 0.1 = small, *r* = 0.3 = medium, and *r* = 0.5 = large (36). All statistical analyses were conducted using IBM SPSS Statistics 25.

## Results

### Descriptive Data

Demographic characteristics of the sample are presented in Table 1 and descriptive statistics for days to return to school and sports are presented in Table 2. Among the full sample, women (median = 5 days) took slightly longer than men (median = 4 days) to return to school (M-W U: *U* = 67,633.5, *p* = 0.001), a small magnitude effect (*r* = −0.11) Women and men did not differ on time to return to sports (median for men: 13 days, women = 14 days, *U* = 55,556.0, *p* = 0.32, *r* = −0.04, negligible effect). Students' age was not associated with days to return to school (Spearman's rho = −0.05, *p* = 0.17). Age was significantly associated with days to return to sports, representing a small magnitude effect (rho = −0.12, *p* = 0.002), although the acute symptom groups did not differ by age [ANOVA: *F*_(2, 805)_ = 2.62, *p* = 0.07]. There were no differences between the acute symptom burden groups in the proportion of men and women [$\chi_{\left(1\right)}^{2}$ = 0.31, *p* = 0.86]. Acute symptom groups did not differ in terms of proportions with self-reported migraine histories, $\chi_{\left(2\right)}^{2}$ = 0.51, *p* = 0.78, but differed in terms of proportions with a self-reported history of depression, $\chi_{\left(2\right)}^{2}$ = 8.89, *p* = 0.01. Days to return to school was significantly positively correlated with days to return to sports (rho = 0.52, *p* < 0.001).

**Table 1:** Summary of demographic and health history information between acute symptom groups.

	**Low Acute Symptom Severity**	**Medium Acute Symptom Severity**	**High Acute Symptom Severity**	**Total sample**
	**(*n* = 264)**	**(*n* = 266)**	**(*n* = 278)**	**(*n* = 808)**
Age, mean (SD), years	20.4 (1.3)	20.6 (1.6)	20.4 (1.3)	20.4 (1.4)
Female gender (*n*, %)	110 (41.7)	116 (43.6)	115 (41.4)	341 (42.2)
Number of prior concussions, mean (SD)	0.9 (1.2)	0.7 (1.0)	0.9 (1.2)	0.8 (1.1)
Zero prior concussions (*n*, %)	136 (51.5)	146 (54.9)	142 (51.1)	424 (52.5)
1 prior concussion (*n*, %)	71 (26.9)	72 (27.1)	70 (25.2)	213 (26.4)
2 prior concussions (*n*, %)	29 (11.0)	32 (12.0)	33 (11.9)	94 (11.6)
3 or more prior concussion (*n*, %)	28 (10.7)	16 (6.1)	33 (11.9)	77 (9.5)
ADHD history (*n*, %)	20 (7.6)	18 (6.8)	27 (9.7)	65 (8.0)
Migraine history (*n*, %)	18 (6.8)	16 (6.0)	21 (7.6)	55 (6.8)
Depression history (*n*, %)	20 (7.6)	15 (5.6)	35 (12.6)	70 (8.7)

*The symptom groups were as follows: Low Acute Symptom Severity (PCSS score 0 through 9), Medium Acute Symptom Severity (10 through 22 for women and 10 through 23 for men), and High Acute Symptom Severity (23 or greater for women and 24 or greater for men). ADHD, attention-deficit/hyperactivity disorder*.

**Table 2:** Number of days to return to school and sports by acute symptom severity.

	**Days to return to school**							**Days to return to sports**						
	***N***	***M***	***Md***	***SD***	***IQR***	**Range**	**Days until 90% return**	***N***	***M***	***Md***	***SD***	***IQR***	**Range**	**Days until 90% return**
Total sample	800	6.5	4	8.0	2–9	0–89	13	690	16.7	14	11.5	10–20	4–83	28
Low Acute Symptom Severity	264	4.1	3	4.7	2–5	0–47	9	238	13.2	11	8.7	8–16	4–79	23
Medium Acute Symptom Severity	263	6.7	5	9.1	2–9	0–89	12	230	17.6	15	12.4	10.75–20	5–83	28
High Acute Symptom Severity	273	8.8	6	8.6	4–11	0–66	17	222	19.4	16	12.2	12–22	6–80	34

*M, mean; Md, median; SD, standard deviation; IQR, interquartile range. The symptom groups were as follows: Low Acute Symptom Severity (PCSS score 0 through 9), Medium Acute Symptom Severity (10 through 22 for women and 10 through 23 for men), and High Acute Symptom Severity (23 or greater for women and 24 or greater for men). Days to return to school is defined as returning without any form of accommodations, not total time absent from school*.

Date of return to school was not available for eight participants (1.0%) and date of return to sports was not available for 118 participants (14.6%). Rates of missing outcome data did not differ between women and men [missing school return data: women = 0.9%, men = 1.1%, $\chi_{\left(1\right)}^{2}$ = 0.08, *p* = 0.78; missing sports return data: women = 13.5%, men = 15.5%, $\chi_{\left(1\right)}^{2}$ = 0.61, *p* = 0.44] and there were no significant differences in rates of missing school return outcome data based on acute symptom burden [missing school return data: Low Acute Symptoms = 0%, Medium Acute Symptoms = 1.1%, High Acute Symptoms = 1.8%, $\chi_{\left(2\right)}^{2}$ = 4.55, *p* = 0.10] but there were differences in rates of missing sports return data [Low Acute Symptoms = 9.8%, Medium Acute Symptoms = 13.5%, High Acute Symptoms = 20.1%, $\chi_{\left(2\right)}^{2}$ = 11.87, *p* = 0.003].

### Return to School

**Figure 1:**
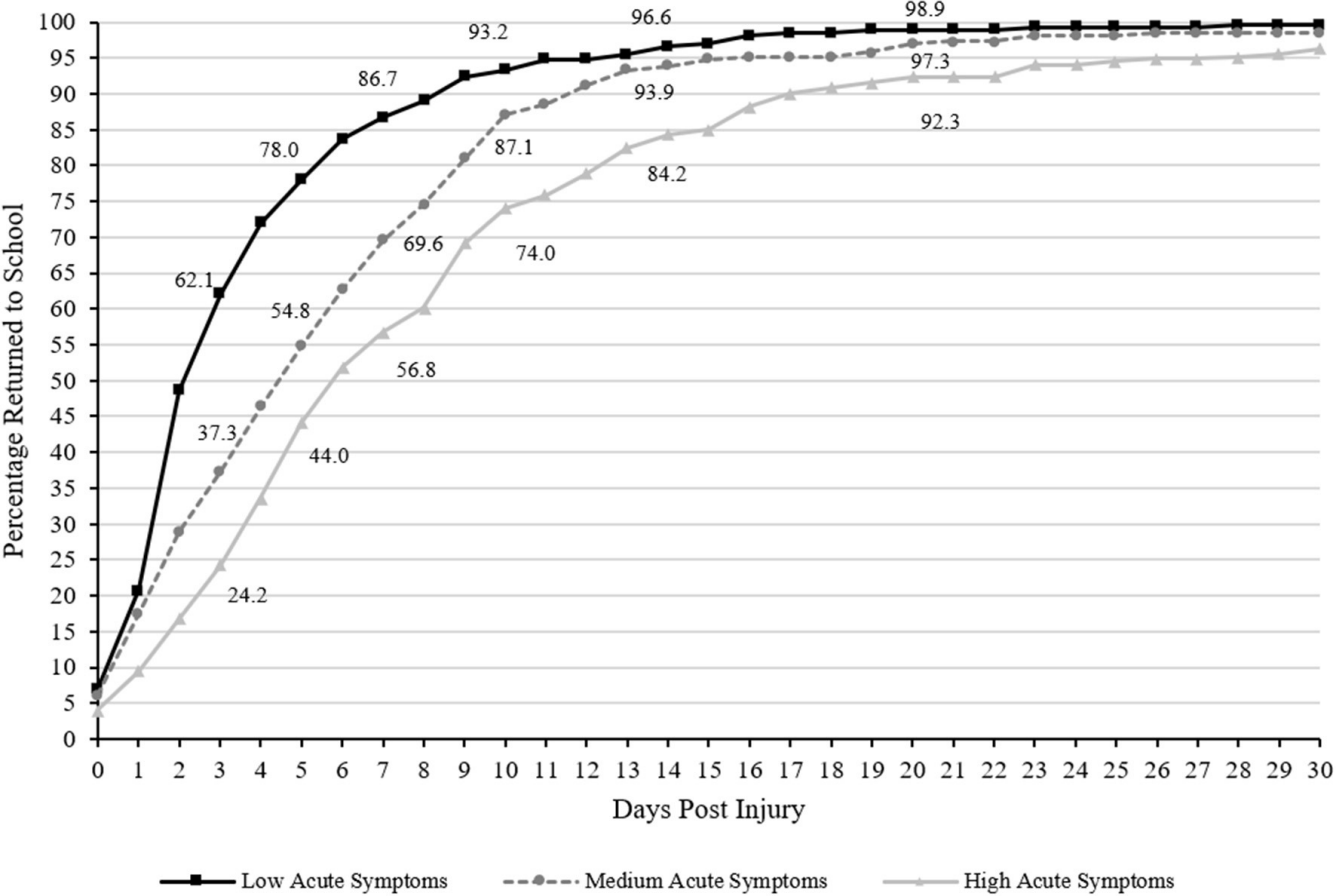
Days to Return to School (cumulative percentage curves). Cumulative percentage curves by group of time to return to school. The featured percentages are as follows: 3 days (24.2% of those with high acute symptom severity, 37.3% of those with medium acute symptom severity, 62.1% of those with low acute symptom severity), 5 days (44% of those with high acute symptom severity, 54.8% of those with medium acute symptom severity, 78% of those with low acute symptom severity), 7 days (56.8% of those with high acute symptom severity, 69.6% of those with medium acute symptom severity, 86.7% of those with low acute symptom severity), 10 days (74% of those with high acute symptom severity, 87.1% of those with medium acute symptom severity, 93.2% of those with low acute symptom severity), 14 days (84.2% of those with high acute symptom severity, 93.9% of those with medium acute symptom severity, 96.6% of those with low acute symptom severity), and 21 days post injury (92.3% of those with high acute symptom severity, 97.3% of those with medium acute symptom severity, 98.9% of those with low acute symptom severity). Days to return to school is defined as returning without any form of accommodations, not total time absent from school.

The Spearman correlation between acute symptoms and days to return to school was rho = 0.37, *p* < 0.001. Cumulative recovery curves displaying the proportion of athletes who returned to school over time based on acute symptoms are presented in Figure 1. Visually it is apparent that the group with low acute symptoms returned more quickly than the other two groups and the medium acute symptom group returned more quickly than the high acute symptom burden group. Statistically, acute symptom groups differed significantly in days to return to school [K-W: $\chi_{\left(2\right)}^{2}$ = 87.50, *p* < 0.001; Log Rank: $\chi_{\left(2\right)}^{2}$ = 89.08, *p* < 0.001]. Follow-up pairwise comparisons revealed a stepwise effect, such that the high acute symptom group (median = 6 days) took significantly longer to return to school than the moderate symptom group (median = 5 days; *U* = 28,714.5, *p* < 0.001, *r* = −0.17, small effect) and the low acute symptom group (median = 3 days; *U* = 19,425.5, *p* < 0.001, *r* = −0.40, medium-large effect) and the moderate acute symptom group took longer to return to school than the low acute symptom group (*U* = 25,413.5, *p* < 0.001, *r* = −0.23, small effect).

There were statistically significant group differences in the proportion of athletes who remained out of school at 3, 5, 7, 10, 14, 21, and 28 days following injury (all *p*'s <0.003; see Table 3 for chi-square values and associated *p-*values). Pairwise chi-square follow-up tests revealed that a greater proportion of athletes with high acute symptoms remained out of school at 3 (OR = 5.14, 95% CI 3.55–7.46), 5 (OR = 4.53, 95% CI 3.11–6.60), 7 (OR = 4.98, 95% CI 3.24–7.65), 10 (OR = 4.80, 95% CI 2.77–8.32), 14 (OR = 5.30, 95% CI 2.53–11.11), 21 (OR = 7.25, 95% CI 2.14–24.61), and 28 days (OR = 13.15, 95% CI 1.71–101.25) compared to those with low acute symptoms. A greater proportion of athletes with medium acute symptoms remained out of school at 3 (OR = 2.76, 95% CI 1.94–3.93), 5 (OR = 2.94, 95% CI 2.01–4.29), 7 (OR = 2.86, 95% CI 1.84–4.45), and 10 days (OR = 2.03, 95% CI 1.12–3.70) compared to those with low acute symptoms, but did not differ significantly at 14 (OR = 1.84, 95% CI 0.80–4.23), 21 (OR = 2.38, 95% CI 0.61–9.30), or 28 days (OR = 4.06, 95% CI 0.45–36.59). Lastly, a greater proportion of athletes with high acute symptoms remained out of school at 3 (OR = 1.86, 95% CI 1.28–2.71), 5 (OR = 1.54, 95% CI 1.10–2.17), 7 (OR = 1.74, 95% CI 1.22–2.49), 10 (OR = 2.37, 95% CI 1.51–3.71), 14 (OR = 2.89, 95% CI 1.58–5.27), 21 (OR = 3.05, 95% CI 1.27–7.30), and 28 days (OR = 3.24, 95% CI 1.04–10.06) compared to those with medium acute symptoms.

**Table 3:** Chi-squared analyses comparing the percentage of athletes who returned to school and sports at various time points post injury based on acute symptom burden.

	**Low Acute Symptom Severity (*n* = 264)**	**Medium Acute Symptom Severity (*n* = 263)**	**High Acute Symptom Severity (*n* = 273)**	**χ^2^**	***p***
	**%**	**%**	**%**		
**Return to school**					
3 days	62.1	37.3	24.2	82.15	<0.001
5 days	78.0	54.8	44.0	66.88	<0.001
7 days	86.7	69.6	56.8	58.70	<0.001
10 days	93.2	87.1	74.0	39.79	<0.001
14 days	96.6	93.9	84.2	29.23	<0.001
21 days	98.9	97.3	92.3	17.04	<0.001
28 days	99.6	98.5	95.2	12.67	0.002
	**Low Acute Symptom Severity** **(*****n*** **=** **238)**	**Medium Acute Symptom Severity** **(*****n*** **=** **230)**	**High Acute Symptom Severity** **(*****n*** **=** **222)**	**χ^2^**	***p***
	**%**	**%**	**%**		
**Return to sports**					
7 days	24.4	7.0	3.6	55.30	<0.001
10 days	47.5	24.8	18.0	52.29	<0.001
14 days	71.0	48.7	41.4	44.43	<0.001
21 days	89.5	78.7	73.9	19.17	<0.001
28 days	95.8	90.4	85.1	15.31	<0.001

*The symptom groups were as follows: Low Acute Symptom Severity (PCSS score 0 through 9), Medium Acute Symptom Severity (10 through 22 for women and 10 through 23 for men), and High Acute Symptom Severity (23 or greater for women and 24 or greater for men). Days to return to school is defined as returning without any form of accommodations, not total time absent from school*.

### Return to Sports

**Figure 2:**
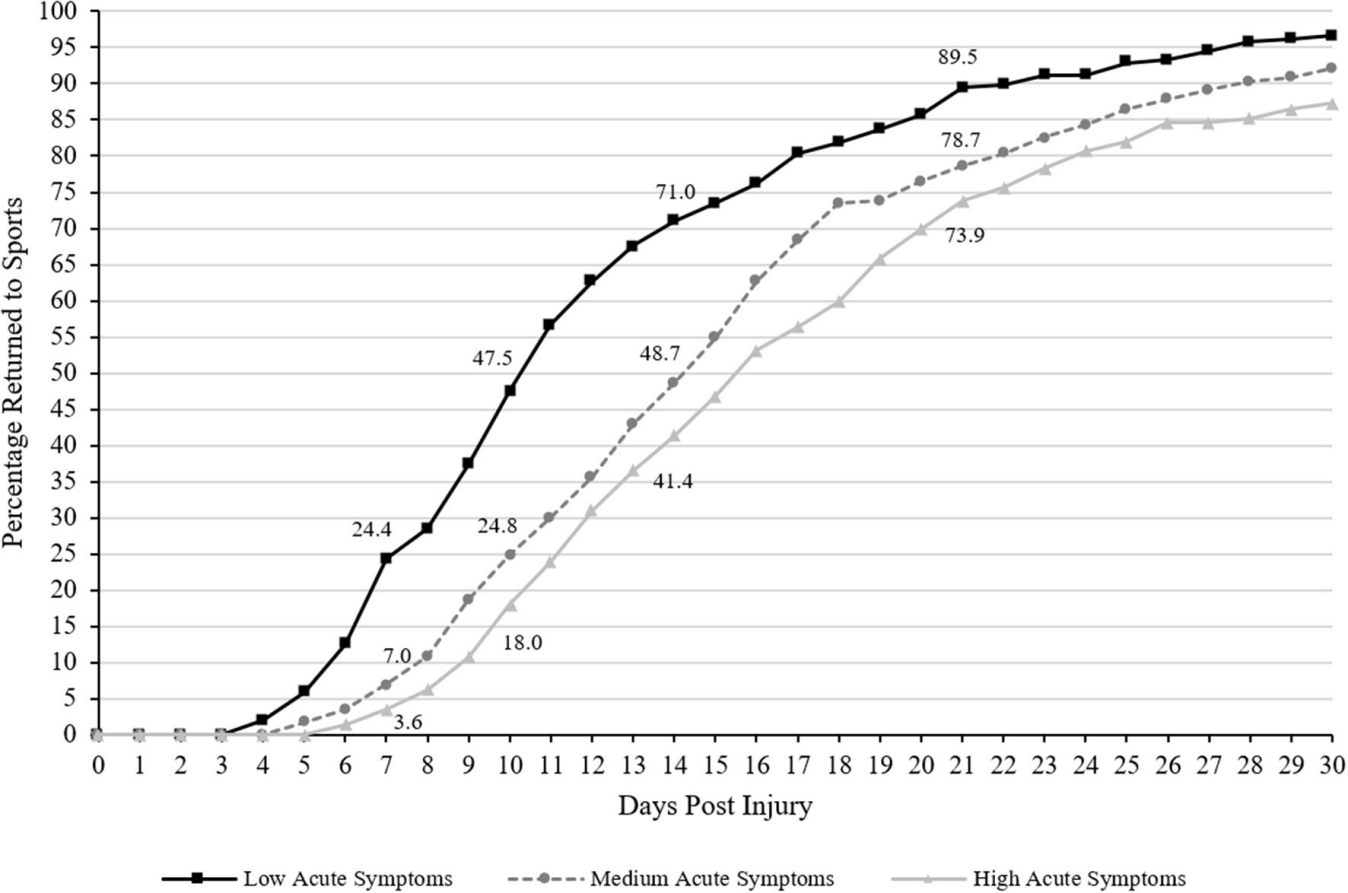
Days to Return to Sports (cumulative percentage curves). Cumulative percentage curves by group of time to return to sports. The featured percentages are as follows: 7 days (3.6% of those with high acute symptom severity, 7% of those with medium acute symptom severity, 24.4% of those with low acute symptom severity), 10 days (18% of those with high acute symptom severity, 24.8% of those with medium acute symptom severity, 47.5% of those with low acute symptom severity), 14 days (41.4% of those with high acute symptom severity, 48.7% of those with medium acute symptom severity, 71% of those with low acute symptom severity), and 21 days post injury (73.9% of those with high acute symptom severity, 78.7% of those with medium acute symptom severity, 89.5% of those with low acute symptom severity).

The Spearman correlation between acute symptoms and days to return to sports was rho = 0.34, *p* < 0.001. Cumulative recovery curves displaying the proportion of athletes who returned to sports over time based on acute symptom burden are presented in Figure 2. Visually it is apparent that the group with low acute symptoms returned more quickly than the other two groups and the medium acute symptom group returned more quickly than the high acute symptom burden group. There were statistically significant differences for days to return to sports between acute symptom groups [K-W: $\chi_{\left(2\right)}^{2}$ = 66.13, *p* < 0.001; Log Rank: $\chi_{\left(2\right)}^{2}$ = 51.69, *p* < 0.001]. Follow-up pairwise comparisons revealed a stepwise effect, such that the high acute symptom (median = 16 days) group took significantly longer to return to sports than the moderate symptom group (median = 15 days; *U* = 22,396.5, *p* = 0.02, *r* = −0.11, small effect) and the low acute symptom group (median = 11 days; *U* = 15,402.0, *p* < 0.001, *r* = −0.36, medium effect) and the moderate acute symptom group took longer to return to sports than the low acute symptom group (*U* = 18,901.5, *p* < 0.001, *r* = −0.27, small-medium effect).

There were statistically significant group differences in the proportion of athletes who remained out of sports at 7, 10, 14, 21, and 28 days following injury (all *p*'s < 0.001; see Table 3 for chi-square values and associated *p*-values). Pairwise chi-square follow-up tests revealed that a greater proportion of athletes with high acute symptoms remained out of sports at 7 (OR = 8.62, 95% CI 4.01–18.53), 10 (OR = 4.11, 95% CI 2.69–6.30), 14 (OR = 3.46, 95% CI 2.35–5.10), 21 (OR = 3.01, 95% CI 1.81–5.02), and 28 days (OR = 3.98, 95% CI 1.91–8.29) compared to those with low acute symptoms. A greater proportion of athletes with medium acute symptoms remained out of sports at 7 (OR = 4.31, 95% CI 2.39–7.76), 10 (OR = 2.74, 95% CI 1.85–4.06), 14 (OR = 2.58, 95% CI 1.76–3.78), 21 (OR = 2.31, 95% CI 1.37–3.88), and 28 days (OR = 2.41, 95% CI 1.12–5.21) compared to those with low acute symptoms. However, there were no significant differences between the proportions of athletes with high acute symptoms that remained out of sports at 7 (OR = 2.00, 95% CI 0.84–4.77), 10 (OR = 1.50, 95% CI 0.95–2.36), 14 (OR = 1.34, 95% CI 0.93–1.95), 21 (OR = 1.31, 95% CI 0.85–2.02), or 28 days (OR = 1.65, 95% CI 0.93–2.93) compared to those with medium acute symptoms.

## Discussion

This study shows a strong association between having a high burden of acute post-concussion symptoms and having a slower return to school and sports in NCAA Division III collegiate athletes. Moreover, it also illustrates the converse: that those athletes with a *low* burden of acute symptoms have a *faster* return to school and sports. This study is unique and important because it is large, includes data collected prospectively over several years using an injury surveillance system deployed to 11 colleges, and it includes return to school as a primary outcome. The vast majority of prior studies on this topic have used subjectively experienced post-concussion symptoms (7, 8, 10–14, 18–20, 22–25) or return to sports as primary outcomes (4, 10, 15–17, 28–30). In particular, previously published studies of injured college students, and studies with combined high school and college student samples, have focused on symptom reporting as the primary outcome (7, 12–14, 25).

Examining time to return to school, arguably, is the most important functional outcome for student athletes, and it has rarely been studied in the sport concussion literature (33). A recently published study from investigators examining data from the Ivy League-Big Ten Epidemiology of Concussion Study found that the median time to symptom resolution was 9 days (IQR = 4–18), return to academics was 8 days (IQR = 3–15), and return-to-full activity was 16 days (IQR = 10–29) (37). There were significant differences between sexes for median time to symptom resolution (one day faster for men) and return to academics (2 days faster for men). In comparison, in the present study the median time to return to school was 4 days (IQR = 2–9), half that reported in the Ivy League-Big Ten study. The reasons for this are unknown and might reflect differences in how athletic trainers were coding the variable—but this also could reflect differences in how students in the Ivy League schools are medically managed. In the present study, at 3 days post injury, only one in four of those with a *high* burden of acute symptoms had fully returned to school (24.2%), and at 5 days two out of five had returned (44.0%). In contrast, for those with a *low* burden of symptoms, roughly two out of three students had fully returned to school by 3 days (62.1%), and roughly three out of four had returned by 5 days (78.0%). Understanding the rates at which student athletes return to school and sports can facilitate future medical management of concussed college athletes, prognostication, and guiding effective and durable return to academics.

### Medical Management

It is recommended that student athletes begin mobilizing, following injury, after a brief period of rest (e.g., 1–2 days) (38). The idea is for them to become gradually more active without significantly exacerbating symptoms, and it is reasonable to avoid vigorous exertion during this early recovery period (38). Those with the highest burden of acute symptoms might have greater difficulty mobilizing and becoming active, and thus they might be more inclined to engage in strict rest or prolonged rest. However, strict rest, for multiple days in the first week following injury is not associated with better outcomes (39). Early mobilization and activity might be beneficial, and there is evolving evidence indicating that exercise can be used relatively early in the recovery process as rehabilitation for those who do not recover swiftly (40–43).

Student athletes are encouraged to begin a graduated return to play protocol after they feel better. This has been termed being asymptomatic at rest (38, 44), although being asymptomatic is difficult to define (45). A recently published study from the NCAA–Department of Defense Concussion, Assessment, Research, and Education (CARE) Consortium reported that starting the graduated return to play protocol while being significantly symptomatic is associated with taking longer to complete the protocol, but not necessarily taking longer to return to play (4). It is not clear, at this point in time, whether a student athlete really needs to be completely “asymptomatic” before becoming more physically active. In fact, the accumulating literature would suggest that *light aerobic exercise* could be started (under supervision) while the student athlete still has symptoms. More research in this area is needed to guide future practice recommendations.

There might be important resource allocation and structural differences in how student athletes are medically managed that might contribute to faster or slower return to school and to sports. For example, NCAA athletes participating in contact and collision sports might have more available and efficient access to medical monitoring and specialty care than athletes in non-contact sports. Similarly, some NCAA athletes from Division I schools might experience more pressure to return to sports more swiftly, given their athletic identity and team pressures, than some athletes from Division III schools. And, of course, NCAA athletes in general might have better access to medical monitoring and specialty health care than high school students. There is some research to suggest that presenting for clinical care later is associated with a longer duration of clinical symptoms and problems (46). Moreover, certain clinical characteristics at the time of the first appointment, such as visual motion sensitivity (a visual-vestibular problem), might be risk factors for prolonged recovery (46). Thus, earlier treatment and rehabilitation might be beneficial for reducing the duration of clinical symptoms and problems.

## Limitations

This study has three important limitations. First, these injury surveillance data were collected during routine clinical care, by athletic trainers, thus there is variability in how concussions were defined/diagnosed. There is no way to determine the accuracy of the concussion diagnoses. Second, there was likely variability in how school personnel defined return to school and return to sports. There was training and ongoing technical support provided to the athletic trainers to ensure these data were captured correctly and reliably. However, it is possible that some of the return to school data represented when the student returned in any capacity, not when they were fully returned to school *without accommodations*. Finally, we examined time to return to school and sports, but not the durability of that return. Data were not available regarding specific academic accommodations provided, or the success or problems encountered during return to school or sports, which represent important areas for future research, and thus we were unable to analyze or adjust for such factors.

## Conclusion

This study shows clearly that having a high burden of acute symptoms, measured in the first 72 h following injury, is associated with taking longer to return to school and sports. For example, at 7 days following injury, 56.8% of athletes who had a high burden of acute symptoms, compared to 86.7% of athletes with a low burden of acute symptoms, had returned to school fully without accommodations. Being unable to attend classes, or participating in school with reduced capacity, can be highly stressful for college students. Given the pace of courses, students can quickly fall behind. Moreover, some student athletes might have been struggling to keep up with their coursework before their injury due to the demands of practicing and competing in their sport—and falling further behind could jeopardize their academic standing and potentially their eligibility as a student athlete. *Personalized precision prognostication* is on the horizon, hopefully within our view. Having a reasonably accurate prognostic model would allow injured student athletes, families, school personnel, and coaches to medically manage the injury better, predict benchmarks for recovery, and implement targeted treatment and rehabilitation (47), as needed, in a sequential approach designed to maximize clinical recovery. More research is needed to better understand factors relating to return to school following concussion, and, most importantly, how health care providers and school personnel can promote a swift, effective, and durable return to the academic demands of college.

## Data Availability Statement

All statistical analyses, and a minimum de-identified dataset, are available to qualified researchers for the purpose of reviewing the results of this study, upon reasonable request. Requests should be directed to Grant L. Iverson, giverson@mgh.harvard.edu.

## Ethics Statement

The studies involving human participants were reviewed and approved by Colby College Institutional Review Board. Written informed consent from the participants' legal guardian/next of kin was not required to participate in this study in accordance with the national legislation and the institutional requirements.

## Author Contributions

GI helped conceptualize the study, conceptualize the statistical analyses, assisted with the literature review, drafted portions of the manuscript, and approved the final manuscript. DT helped with the literature reviews, reviewed the statistical analyses, edited the manuscript, and approved the final manuscript. BM helped develop the injury surveillance application, design and coordinate data collection, and approved the final manuscript. RZ critically reviewed, edited, and approved the final manuscript. PB helped design and coordinate data collection, wrote the IRB, conceptualized the injury surveillance program, investigated database questions, and edited and approved the final manuscript. NC helped conceptualize the study, conceptualize the statistical analyses, conducted the statistical analyses, drafted portions of the manuscript, edited the manuscript, and approved the final manuscript. All authors approved the final manuscript as submitted and agree to be accountable for all aspects of the work.

## Funding

This study was funded in part by the National Football League for a program of research entitled The Spectrum of Concussion: Predictors of Clinical Recovery, Treatment and Rehabilitation, and Possible Long-Term Effects (PI: GI). The authors acknowledge prior funding from the Goldfarb Center for Public Policy and Civic Engagement at Colby College, and the Bill and Joan Alfond Foundation (PI: PB). NC acknowledges support from the Louis V. Gerstner III Research Scholar Award. GI acknowledges unrestricted philanthropic support from the Mooney-Reed Charitable Foundation, Heinz Family Foundation, Boston Bolts, ImPACT® Applications, Inc., and the Spaulding Research Institute. The above entities were not involved in the study design, collection, analysis, interpretation of data, the writing of this article or the decision to submit it for publication.

## Conflict of Interest

GI serves as a scientific advisor for NanoDX®, Sway Operations, LLC, and Highmark, Inc. He has a clinical and consulting practice in forensic neuropsychology, including expert testimony, involving individuals who have sustained mild TBIs (including former athletes), and on the topic of suicide. He has received research funding from several test publishing companies, including ImPACT Applications, Inc., CNS Vital Signs, and Psychological Assessment Resources (PAR, Inc.). He has received research funding as a principal investigator from the National Football League, and subcontract grant funding as a collaborator from the Harvard Integrated Program to Protect and Improve the Health of National Football League Players Association Members. DT is a consult for REACT Neuro, Inc. RZ receives royalties from Oakstone for an educational CD (Physical Medicine and Rehabilitation: A Comprehensive Review) and from demosMedical, part of Springer Publishing, for serving as co-editor of the text Brain Injury Medicine and serves on the Scientific Advisory Board of Oxeia Biopharma, BioDirection, ElMINDA, and Myomo. He is also PI on a grant entitled the Football Players Health Study at Harvard University, which is funded by the National Football League Players Association (NFLPA) and evaluates patients for the MGH Brain and Body TRUST Center, sponsored in part by the NFLPA, and serves on the Mackey-White Health and Safety Committee. The remaining authors declare that the research was conducted in the absence of any commercial or financial relationships that could be construed as a potential conflict of interest.

## Publisher's Note

All claims expressed in this article are solely those of the authors and do not necessarily represent those of their affiliated organizations, or those of the publisher, the editors and the reviewers. Any product that may be evaluated in this article, or claim that may be made by its manufacturer, is not guaranteed or endorsed by the publisher.
